# *Tremella fuciformis* Crude Polysaccharides Attenuates Steatosis and Suppresses Inflammation in Diet-Induced NAFLD Mice

**DOI:** 10.3390/cimb44030081

**Published:** 2022-03-03

**Authors:** Tariq Jamal Khan, Xiaofei Xu, Xiaoling Xie, Ximing Dai, Pingnan Sun, Qingdong Xie, Xiaoling Zhou

**Affiliations:** 1 Guangdong Provincial Key Laboratory of Infectious Diseases and Molecular Immunopathology, Shantou University Medical College, Shantou 515041, China; tariqstu1@gmail.com (T.J.K.); xxling1995@163.com (X.X.); 19xmdai@stu.edu.cn (X.D.); pnsun@stu.edu.cn (P.S.); qdxie@stu.edu.cn (Q.X.); 2 Geometry Cell Biology Research Center, Dongguan 523808, China; xfxu@scici.com; 3 The Center for Reproductive Medicine, Shantou University Medical College, Shantou 515041, China

**Keywords:** *Tremella fuciformis* crude polysaccharides, nonalcoholic fatty liver disease, methionine–choline-deficient diet

## Abstract

Nonalcoholic fatty liver disease (NAFLD) is a chronic liver disorder characterized by an enhanced accumulation of lipids, which affects around 40% of the world’s population. The *T. fuciformis* fungus possesses immunomodulatory activity and other beneficial properties that may alleviate steatosis through a different mechanism. The present study was designed to evaluate the effect *T. fuciformis* crude polysaccharides (TFCP) on inflammatory and lipid metabolism gene expression, oxidative stress, and lipid profile. Mice were divided into groups receiving (a) a normal chow diet (NCD), (b) a methionine–choline-deficient (MCD) diet, and (c) a MCD diet with TFCP. Liver histopathology was performed, and the hepatic gene expression levels were estimated using qRT-PCR. The lipid profiles, ALT, AST, and efficient oxidative enzymes were analyzed using ELISA. The TFCP administration in the MCD-fed mice suppressed hepatic lipid accumulation, lipid metabolism-associated genes (HMGCR, FABP, SREBP, ACC, and FAS), and inflammation-associated genes (IL-1β, TLR4, TNF-α, and IL-6) whilst enhancing the expression of HNF4α genes. TFCP mitigated against oxidative stress and normalized healthy lipid profiles. These results highlighted that TFCP prevents NAFLD through the inhibition of oxidative stress and inflammation, suggesting TFCP would potentially be an effective therapeutic agent against NAFLD progression.

## 1. Introduction

Nonalcoholic fatty liver disease (NAFLD) is a severe hepatic disorder causing a huge overall burden of liver diseases [1]. NAFLD may be defined as the umbrella term covering a range of abnormalities, from NAFLD to nonalcoholic steatohepatitis (NASH), which may progress to fibrosis and cirrhosis [1]. NAFLD is closely associated with metabolic syndrome. The abnormalities that develop in NAFLD patients include (i) abdominal obesity, (ii) high blood pressure, (iii) high blood glucose, (iv) high serum triglycerides, and (v) a low abundance of serum high-density lipoprotein cholesterol (HDL-C), which was reported to contribute significantly to the disease [2]. The ethnicity of the patients influences NAFLD development [3]. In in vivo models, the methionine–choline-deficient (MCD) diet was reported to induce steatohepatitis; however, it does not influence body weight gain and obesity [4]. Additionally, a high occurrence of NAFLD was observed in obese and diabetic patients, while non-diabetic and lean NAFLD patients were also reported to a lesser extent [5].

To date, a huge amount of research has evidenced the crucial role of toll-like receptor 4 (TLR4) and dysbiosis in NAFLD development [5]. Indeed, gut microbiota (GM) have been found to extend their axes bi-directionally (i.e., endocrine and immunological pathways) with numerous extra-intestinal organs, such as the brain, kidneys, and bone [4]. Nevertheless, this gut–liver axis plays an essential role during the progression of chronic liver diseases, such as hepatitis B and C, NAFLD, NASH, and hepatocellular carcinoma (HCC) [6]. GM-induced dysbiosis enhances the hepatic TLR4 expression and pro-inflammatory cytokine levels in NAFLD and NASH patients by disrupting the intestine’s inner lining and prompting the lipopolysaccharide (LPS) translocation in TLR4 signaling activation [7]. Thus, LPS–TLR4 pathways in NAFLD pathogenesis could be marked as a significant cause of NASH [5].

*T. fuciformis* is a well-known edible mushroom and was reportedly used in ancient Chinese society as a traditional medicine [8,9]. The *T. fuciformis* fungus possesses antitumor, anti-inflammatory, and immunomodulatory properties [10]. *T. fuciformis* administration has been reported to significantly improve the serum IL-2, IL-12, INF-γ, and IgG levels in cyclophosphamide-induced immunosuppressed mice and show immunomodulatory abilities [8]. Additionally, the *T. fuciformis* fungus may alleviate colonic tissue damage in colitic mice by shortening the length of the colon and lowering the concentration of D-lactate [10].

The hepatocyte nuclear factor 4α (HNF4α, *NR2A1*) is a member of the superfamily of nuclear hormone receptors that are expressed in the liver, kidney, pancreatic islets, and gut and is engaged in regulating the gene expression involved in fatty acid, cholesterol, glucose, and urea synthesis [11]. HNF4α plays a significant role in lowering inflammation, and the reduced expression of HNF4α was observed in alcoholic and nonalcoholic steatohepatitis patients [12]. In contrast, the overexpression of HNF4α has been shown to promote a substantial anti-inflammatory effect in mesenchymal stem cells (MSC) through a nuclear factor kappa B (NF-κB)-dependent pathway [13].

Considering the above background, this study was designed to investigate the effects of TFCP on intrinsic immunogenic and metabolic activities in methionine–choline-deficient (MCD) mice with diet-induced NAFLD. Here, we studied the impact of TFCP on the serum parameters, including lipid profile, antioxidants, liver enzymes (ALT and AST), and the gene expression of inflammatory cytokines (IL-1β, HNF4α, TLR4, TNF-α, and IL-6) and lipid metabolism-associated genes (HMGCR, FABP, SREBP, ACC, and FAS) in the MCD-diet-induced NAFLD mice. Our results indicated that TFCP could help to alleviate NAFLD by suppressing inflammation.

## 2. Materials and Methods

### 2.1. TFCP Preparation and Characterization

*T. fuciformis* crude polysaccharides (TFCP), provided by Dr Xiaofei Xu, were obtained from the fruit body of *T. fuciformis* by boiling water extraction. Dry *T. fuciformis* fruiting bodies were collected from Gutian, Fujian province, China. The *T. fuciformis* fruiting bodies were crushed and sifted through a 40 mesh prior to use. TFCP was extracted using the hot water extraction method (1:60 *w*/*v*) at 95 °C for 6 h. Then, the solution was filtered and concentrated to quarter of its original weight under vacuum conditions at 70 °C. Deproteination was performed by adjusting the pH of the solution to 4.0 using citric acid and it was then kept at 4 °C for 2 days by isoelectric precipitation. Collecting supernatant and three equivalent volumes of 95% ethanol were added to the precipitate polysaccharides at 4 °C overnight. The TFCP fraction was obtained by centrifugation and lyophilization. The TFCP powder was white in color. The peak molecular weight of the TFCP was about 1100 kDa, as measured using high-performance gel permeation chromatography (HPGPC). The saccharide content of the TFCP was 82%, as detected by phenol sulfuric acid analysis with mannose as a standard. Protein content was measured by the Bradford method. The protein content was 2.4% in the TFCP fraction. Fourier transform infrared (FT-IR) spectroscopy of the TFCP samples was measured to be in the range of 4000–400 cm^−1^ by the KBr disk method using an FT-IR spectrometer (Nicolet IS50-Nicolet Continuum, Thermo Fisher Scientific). OMNIC software was used for the spectroscopy analysis. As shown in following figure, the characteristic peaks of FT-IR spectra of the TFCP fraction at approximately 3412, 2935, 1721, 1606, 1424, 1135, 1071, 915, and 803 cm^−1^ are presented. The absorbance at 3412 and 2935 cm^−1^ are due to the stretching of O–H and -CH2 groups, respectively. The peaks at 1721 and 1606 cm^−1^ demonstrate the presence of the uronic acid group. High absorbance in the region of 1200–1000 cm^−1^ is typical for the polysaccharides molecule through the stretching of C–O, C–C, and C–OH. The peak at 915 cm^−1^ is due to the presence of D-glucopyranosyl and the peak at 803 cm^−1^ suggests the presence of α-glycosidic bonds in the molecular structure of the TFCP.

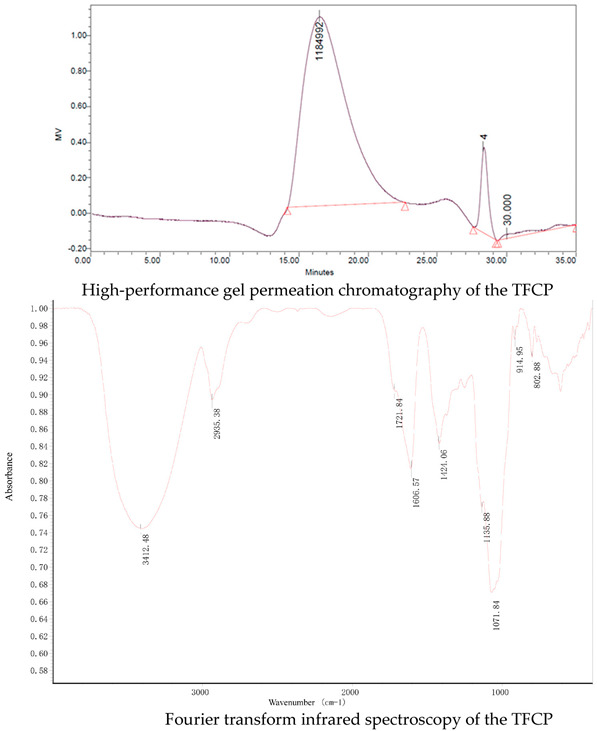



### 2.2. Animal Experiments

For the in vivo experiments, specific pathogen-free (SPF) male Kunming mice (18–22 g) were housed in a controlled environment (23 °C, 12 h daylight cycle, lights off at 18:00 h, and relative humidity was 40–50%). Notably, the Institutional Animal Care and Use Committee of Shantou University Medical College provided the desired approvals and supervision for the animal experiments according to the National Research Council Guide for Care and Use of Laboratory Animals.

Following the standard protocols, all mice were kept for seven days with free access to water and a standard chow diet to acclimatize before treatments. The mice were then divided randomly into three different groups based on diet and/or treatment: the NCD control group (*n* = 5); the methionine–choline-deficient (MCD) diet group (*n* = 5); and the intervention group (MCD + TFCP) (*n* = 5). Mice from the control group were fed the standard diet with free access to water. The MCD diet contained 40% sucrose and 10% fat as ingredients but lacked methionine and choline, which are necessary for hepatic mitochondrial β-oxidation [14]. The MCD and MCD + TFCP groups were fed the methionine–choline-deficient (MCD) diet. The intervention group underwent daily intragastric administration of TFCP (200 mg per kg body weight per day). In contrast, the MCD group received the same amount of normal saline once per day for six weeks [10].

After six weeks of the study, the mice were fasted for 12 h and then we collected blood and tissue samples. All animals were euthanized using pentobarbital sodium.

### 2.3. Collection of Liver Tissue and Their Histopathological Analysis

Mice (treated and untreated) were considered for dissection to collect hepatic tissues for histopathological analysis. Liver samples were excised immediately after sacrifice using a scalpel and placed into appropriate tubes for the histopathologic examinations. For histological analysis, the liver tissues were fixed in 4% paraformaldehyde for 4 h. Paraformaldehyde-fixed paraffin sections of the liver were stained with hematoxylin–eosin (HE) for pathological analysis. The images were captured by an optical microscope (Leica DMI3000B, USA).

### 2.4. Serum and Blood Measurements

Alanine aminotransferase (ALT), aspartate aminotransferase (AST), and high-density lipoprotein (HDL-C) in the serum were quantitated using an automated analyzer (Sysmex CHEMIX-180, Japan). The concentrations of serum triglycerides (TG) and total cholesterol (TC) were separately quantitated using triglyceride and cholesterol assay kits (Applygen Technologies Inc., Beijing, China) according to the manufacturer’s instructions. Additionally, the oxidative stress was elucidated by measuring the malondialdehyde (MDA) and superoxide dismutase (SOD) enzymes in the serum samples following ELISA-based standard protocols.

### 2.5. Real-Time Quantitative Polymerase Chain Reaction

Total RNA was extracted from the liver using TRIzol (D9108B, Takara, Dalian, China) and reverse-transcribed into cDNA using PrimeScript RT master mix (RR036A, Takara, Dalian, China). Real-time quantitative polymerase chain reaction (qRT-PCR) was performed with Applied Biosystems 7500 real-time PCR system using the SYBR Premix Ex Taq (Tli RNase H Plus) (RR420A, Takara, Dalian, China). The primers of the target genes were synthesized by Sangon Biotech (Shanghai, China). Glyceraldehyde 3-phosphate dehydrogenase (GAPDH) (B661304, Sangon Biotech) was used as the internal control.

### 2.6. Statistical Analysis

Data were expressed as the mean ± SEM. Statistical analyses were performed using the student *t*-test in IBM SPSS Statistics 20. *p* < 0.05 was considered statistically significant. Drawn figures were created using GraphPad Prism 8.

## 3. Results

### 3.1. Inhibition of Hepatic Steatosis and Reduction in Liver Weight through TFCP Administration

**Figure 1:**
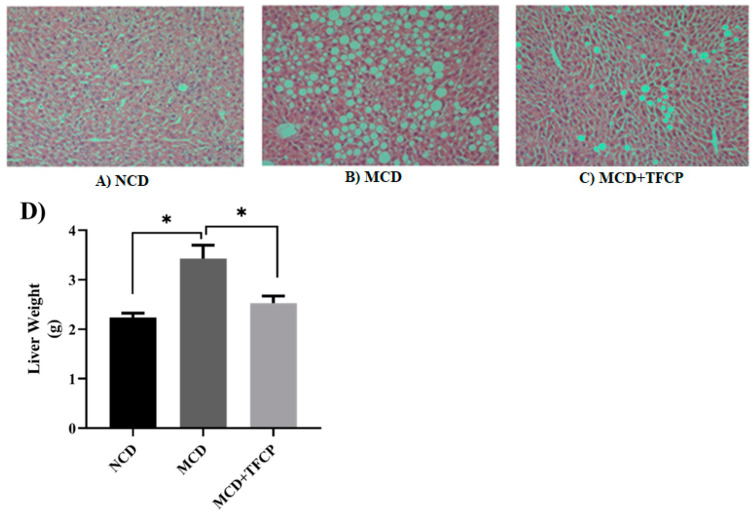
The TFCP-induced attenuation in hepatic lipid deposition and liver weight decline in MCD-fed NAFLD mice. Mice were fed with MCD or the control diet for six weeks, while the intragastric administration of TFCP to MCD-fed mice was performed daily. (**A**–**C**) The histological analysis of the HE stained liver sections from the mice, demonstrating differential lipid deposition in the livers of the mice in the different groups. (**D**) The graph demonstrates the decline in the liver weight of the NCD-, MCD- and MCD + TFCP-treated groups of mice. The means ± SEM of the results in the graph were obtained using NCD (*n* = 5), MCD diet-fed group (*n* = 5), and MCD + TFCP (*n* = 5). Data were shown as the mean ± SEM (* *p* < 0.05).

A comparative study on the lipid deposition in the MCD-induced NAFLD livers of the experimental mice was carried out to assess the anti-NAFLD activity of TFCP through histopathology and liver weight. Briefly, the HE stained micrograph obtained from the livers of MCD-fed mice (Figure 1B) demonstrated a higher lipid droplet density than NCD mice (Figure 1A). In contrast, a decline in lipid droplet density in MCD-fed livers was noticed when treated with TFCP (Figure 1C). The primary histopathology observations were validated by weighing the livers obtained from the treated and untreated groups. The data presented in Figure 1D were found to agree with the histopathology demonstrated in Figure 1A–C. Strictly speaking, compared to MCD-fed mice (3.43 ± 0.268 g), a smaller decline in the liver weight was noted in MCD + TFCP-fed mice (2.26 ± 0.06 g) (Figure 1D). Overall, the liver weight data reflected NCD, MCD, and MCD + TFCP lipid accumulation trends. Thus, it can be advocated that TFCP effectively attenuates NAFLD progression.

### 3.2. Effects of TFCP on Triglyceride (TG), Total Cholesterol (TC), and High-Density Lipoprotein Cholesterol (HDL-C) Levels in the Serum

**Figure 2:**
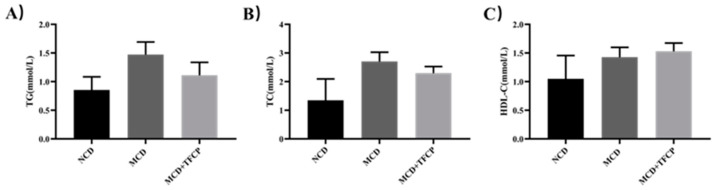
TFCP normalized the altered lipid profile of the studied group. Mice were fed with MCD or the control diet for six weeks, while the intragastric administration of TFCP to the MCD-fed mice was performed daily. Lipid profiles including (**A**) TG, (**B**) TC, and (**C**) HDL-C levels were evaluated in the mice’s serum. The means ± SEM of the results in the graph were obtained using NCD (*n* = 5), MCD diet-fed group (*n* = 5), and MCD + TFCP (*n* = 5). Data were shown as the mean ± SEM.

Briefly, the levels of different lipids, including triglycerides (TG), total cholesterol (TC), and high-density lipoprotein cholesterol (HDL-C), were analyzed from the sera of the MCD- and MCD + TFCP-treated groups. The enhanced TG and TC levels indicated the diet’s effectiveness in developing the NAFLD model [15]. The level of TG was increased in MCD-fed mice (1.47 ± 0.21 mmol/L); however, the TG level was reduced in those fed with MCD + TFCP (1.11 ± 0.22 mmol/L) (Figure 2A). Similarly, the cholesterol-lowering ability of TFCP was reported in the TC profile of the TFCP-treated group. The serum TC level in the MCD-fed group was 2.70 ± 0.32 mmol/L (Figure 2B). However, as expected, under identical experimental conditions, the serum TC level was reduced to 2.30 ± 0.23 mmol/L in the MCD + TFCP group (Figure 2B). Additionally,. an elevation in HDL-C level was noted in MCD+TFCP-treated mice (1.53 ± 0.14 mmol/L) compared to the MCD-fed mice (1.43 ± 0.17 mmol/L) (Figure 2C). The TFCP treatment reduced the concentration of TC and TG while increasing the HDL-C level under NAFLD conditions.

### 3.3. TFCP Administration Reduced the Expression of the Genes Involved in Cholesterol Metabolism and Transport

**Figure 3:**
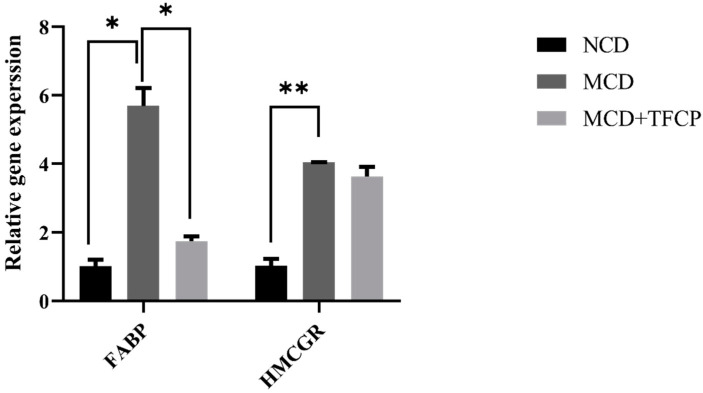
The TFCP-induced suppression in the cholesterol synthesis and transport in MCD-fed mice. Mice were fed with MCD or the control diet for six weeks, while the intragastric administration of TFCP to MCD-fed mice was performed daily. The mRNA levels of FABP and HMCGR genes were measured by qPCR. The means ± SEM of the results in the graph were obtained using NCD (*n* = 5), MCD diet-fed group (*n* = 5), and MCD + TFCP (*n* = 5). Data were shown as the mean ± SEM (** *p* < 0.01, * *p* < 0.05).

To study the underlying mechanism involved in lipid metabolism and transport, we determined the expression levels of 3-hydroxy-3-methyl-glutaryl-coenzyme A reductase (HMGCR rate-limiting enzyme for cholesterol synthesis) and fatty acid-binding protein (FABP is responsible for the transportation and metabolism of fatty acids across cells) genes in the livers of the mice [16,17]. The cholesterol synthesis and its transport across cells are essential in NAFLD and NASH [16,17]. Hence, we also studied the effect of TFCP administration on the regulation of the genes that are responsible for the synthesis and transport of lipid molecules across liver cells. Concisely, the expression of the FABP gene was found to be downregulated 1.74 ± 0.14-fold in the MCD + TFCP-treated group compared to 5.69 ± 0.52-fold in MCD-fed mice (Figure 3). On the other hand, relative to the NCD control, the expression of the HMCGR gene was significantly higher in the MCD-fed group and was quantitated as 4.05 ± 0.01-fold higher (Figure 3). However, compared to the MCD group, we observed the lowered expression of the HMCGR gene in the MCD + TFCP group (3.63 ± 0.29-fold) (Figure 3). Overall, the TFCP treatment suppressed the cholesterol synthesis and transport, thus mitigating against NAFLD.

### 3.4. TFCP Administration Reduces the Expression of Lipid-Metabolism-Associated Genes

**Figure 4:**
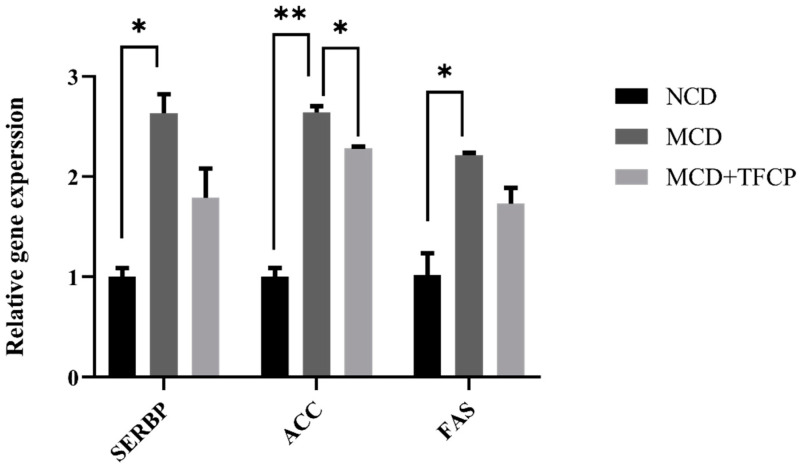
TFCP suppressed the lipid synthesis in the livers of MCD-fed mice. Mice were fed with MCD or the control diet for six weeks, while the intragastric administration of TFCP to MCD-fed mice was performed daily. The mRNA levels of SREBP, ACC, and FAS genes were measured by qPCR. The means ± SEM of the results in the graph were obtained using NCD (*n* = 5), MCD diet-fed (*n* = 5), and MCD + TFCP-treated (*n* = 5) group. Data were shown as the mean ± SEM (** *p* < 0.01, * *p* < 0.05).

To understand the mechanism involved in the reduced lipid levels in TFCP-treated mice, we determined the expression levels of the genes that are involved in lipogenesis in the mice’s livers. Here, we studied the well-known lipid metabolism-associated genes during NAFLD progression [18]. Hepatic lipid synthesis is mainly modulated by sterol regulatory element-binding protein (SREBP), a master regulator of lipid biosynthesis. SREBP regulates the expression of other triglycerides and fatty acid synthesis, mainly Fas cell surface death receptor (FAS) and acetyl-CoA carboxylase (ACC, a biotin-dependent enzyme) [19,20,21]. Overall, the upregulation of the lipid metabolism-associated genes viz. SREBP, ACC, and FAS was noticed in the MCD group (Figure 4). In contrast, a decrease in SREBP, FAS, and ACC expression was observed in MCD + TFCP group (Figure 4). The SREBP expression was found to be 2.63 ± 0.19-fold higher in the MCD-fed group but was reduced 1.79 ± 0.29-fold in the MCD + TFCP-treated group (Figure 4). Similarly, the downregulated expression of the ACC gene was observed in the MCD + TFCP group (2.28 ± 0.02-fold) compared to the MCD (2.64 ± 0.06-fold) group (Figure 4). Additionally, the suppressed expression of the FAS gene was noted in MCD + TFCP group (1.73 ± 0.16-fold) compared to the MCD group (2.21 ± 0.03-fold) (Figure 4). Finally, the TFCP treatment downregulated lipogenesis in the MCD-fed mice.

### 3.5. TFCP Downregulates the Expression of Inflammation-Associated Genes in the Liver

**Figure 5:**
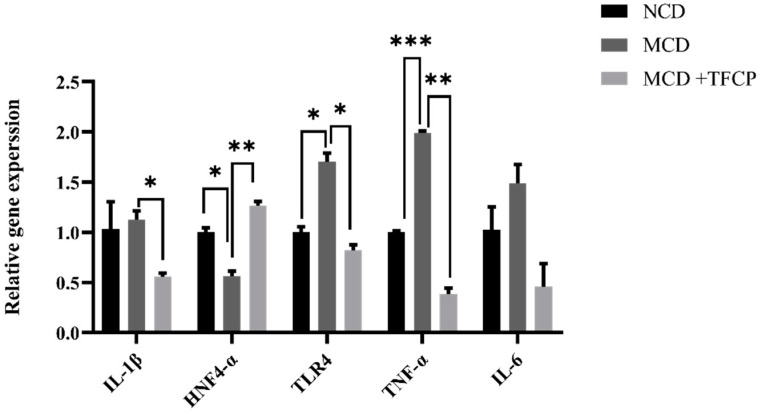
The TFCP-induced downregulation inflammation in MCD-fed mice. Mice were fed with MCD or the control diet for six weeks, while the intragastric administration of TFCP to MCD-fed mice was performed daily. The mRNA levels of IL-1β, HNF4α, TLR4, TNF-α, and IL-6 were measured by qPCR. The means ± SEM of the results in the graph were obtained using NCD (*n* = 5), MCD diet-fed group (*n* = 5), and MCD + TFCP (*n* = 5). Data were shown as the mean ± SEM (*** *p* < 0.001; ** *p* < 0.01, * *p* < 0.05).

Toll-like receptor 4 (TLR4) is a pattern recognition receptor for lipopolysaccharides (LPS) and induces the activation of innate immunity. The TLR4 and pro-inflammatory signaling pathways play a key role in the progression of NAFLD [18]. It was presumed that TFCP inhibits pro-inflammatory cytokines synthesis through the downregulation of TLR4. As expected, TLR4 expression was significantly downregulated by TFCP treatment in the mice fed with MCD. Consistent with the TLR4 expression, the hepatic mRNA expression of pro-inflammatory cytokines, such as IL-1β, TLR4, TNF-α, and IL-6, was also increased in the mice fed with the MCD diet; however, it was reduced by the TFCP treatment (Figure 5). The bar graph presented in Figure 5 demonstrates the alteration of inflammatory genes, namely IL-1β, TLR4, TNF-α, IL-6, and HNF4α. The enhanced expression of IL-1β (a pro-inflammatory cytokine) was observed in the MCD group (1.13 ± 0.09-fold) (Figure 5), whereas in the MCD + TFCP group, the expression of the IL-1β gene was found as 0.56 ± 0.04-fold lower (Figure 5). In MCD-fed mice, the upregulated expression of TLR4 (1.70 ± 0.09-fold), TNF-α (2.00 ± 0.02-fold), and IL-6 (1.49 ± 0.19-fold) genes was noticed (Figure 5). With the treatment of TFCP, the downregulation of the TLR4, TNF-α, and IL-6 genes was noticed in MCD + TFCP group (0.82 ± 0.06-fold, 0.38 ± 0.06, 0.46 ± 0.23, respectively) (Figure 5). HNF4α expression controls the inflammation and associated genes during NAFLD progression [22]. The downregulated expression of HNF4α was noticed in the MCD group (0.56 ± 0.05-fold) (Figure 5). The enhanced expression of HNF4α was found in the MCD + TFCP group (1.27 ± 0.04-fold), reflecting the anti-inflammatory ability of TFCP (Figure 5). TFCP downregulated the expression of pro-inflammatory cytokines while enhancing the expression of HNF4 and blocking inflammation.

### 3.6. Effect of TFCP on the ALT, AST, MDA, and SOD Levels in the Mice Serum

Here, the hepatoprotective ability of TFCP was studied and an increased level of oxidative stress was also reported. The data in Figure S1 highlight the levels of ALT, AST, SOD, and MDA in the serum. In brief, the ALT level in the serum of the NCD-fed mice was 65.88 ± 2.22 U/L and it was 715.18 ± 278.41 U/L in the MCD-fed group; however, the ALT level declined to 74.69 ± 25.16 U/L in the MCD + TFCP group (Figure S1). Similarly, the AST level followed the same trend as ALT and was found to reduce to 143.92 ± 14.79 U/L in the MCD + TFCP group compared to the MCD group (475.39 ± 222.87 U/L) (Figure S1). A reduced MDA level was also observed in the MCD + TFCP group (11.43 ± 0.36 nmol/L) (Figure S1) compared to the MCD- (13.21 ± 0.35 nmol/L) and NCD-fed (10.0 ± 1.07 nmol/L) groups. Additionally, an enhanced SOD level was found in the MCD + TFCP-treated mice (318.64 ± 5.49 U/mL), while levels of 321.38 ± 27.46 U/mL and 341.98 ± 17.85 U/mL were noticed in the MCD and NCD groups, respectively (Figure S1).

## 4. Discussion

Our findings present TFCP as a potential anti-NAFLD compound. The experimental design of this study encompassed an array of state-of-the-art techniques, including (i) the induction of NAFLD in mice by feeding them an MCD diet [23], (ii) the validation of NAFLD development by HE stained visuals based the histopathological analysis of liver tissues, (iii) the bioactivity of critical parameters related to NAFLD, such as: serum ALT, AST, MDA, SOD, and lipid profile; the expressions of lipid-associated genes, such as HMGCR, FABP, SREBP, ACC, and FAS; and the expressions of inflammation-associated genes, including IL-1β, HNF4α, TLR4, TNF-α, and IL-6.

Strictly speaking, one of the most established characteristics of NAFLD hepatic cells is their massive destruction due to lipid infiltration within the hepatic lobule [23]. The micrographs presented in Figure 1 demonstrate the histopathological and morphological states of the HE stained liver sections of the NCD-, MCD- and MCD + TFCP-treated mice.

The enhanced accumulation of hepatic triglyceride in NAFLD may be caused by the increased synthesis of fatty acids and suppressed fatty acid oxidation [24]. The present study demonstrated that TFCP regulated hepatic fatty acid synthesis through the reduced expression of fatty acid synthesis regulators, including SREBP, FAS, and ACC, and also suppressed NAFLD. SREBP is a transcription factor for TG synthesis via the activation of genes that are responsible for fatty acid synthesis and the storage of TG through FAS [25]. In addition, the data presented in this study suggest that the treatment of TFCP exerted SREBP-induced control over cellular lipid metabolism and homeostasis in MCD-fed animals [26]. Similarly, the crucial role of ACC is to convert acetyl-CoA into malonyl CoA and is a rate-controlling step in de novo lipogenesis (DNL) [27]. A high level synthesis of malonyl-CoA is needed to regulate the hepatic mitochondrial fat oxidation [28]. Owing to this, the downregulation of ACC enzymes from TFCP treatments suggests a modulation role of TFCP on DNL process in NAFLD mice. In addition to this, the disturbance in HDL-C levels (Figure 2C) could be assigned to the development of morphological vascular changes and cardiometabolic risk factors, including NAFLD [29]. Interestingly, the enhanced TG and TC levels (Figure 2A,B) could be valuable markers for atherogenic lipid abnormalities, as well as insulin resistance, metabolic syndrome (MetS), and high cardiovascular risk [30,31].

In NAFLD studies, it is well established that hepatic TLR4 is activated by translocated gut-derived LPS [32]. TLR4 leads to the activation of downstream signaling pathways, mainly NF-κB, which further upregulates the expression of pro-inflammatory cytokines, including IL-1β and IL-6 [33]. The deletion of hepatic TLR4 prevents inflammation [34]. Zhu et al. [35] suggested that the translocation of LPS and the subsequent activation of the TLR4 pathway significantly impacts the pathological mechanism of MCD diet-induced NAFLD and NASH mice. In the present study, TFCP was reported to possess the ability to suppress inflammation, and the reduced activation of TLR4 in the MCD + TFCP group was noted. Interestingly, our results agree with previous studies, witnessing the increasing trends in IL-1β, TLR-4, TNF-α, and IL-6 genes in mice subjected to the MCD diet. In contrast, a decreasing trend was noticed in the MCD + TFCP group. Similarly, it has been observed that the green extract of *Penthorum chinese* is a potential immune stimulator and can affect the release of inflammatory factors in serum (NADPH, IL-1β, TNF-α, and IL-6) and the expression of proteins (CYP2E1, IL-1β, TNF-α, and IL-6) in the livers of rats [36].

Evidence has supported the role of HNF4α in controlling lipid metabolism and inflammation in patients using knockout animal models of alcoholic or nonalcoholic steatohepatitis [37]. The ablation of hepatic HNF4α gene expression causes steatosis with a disturbed lipid profile and an upregulated expression of inflammatory genes [38]. HNF4α activation using oligonucleotide therapy represents an approach for treating NAFLD [39]. Here, the administration of TFCP to the MCD-induced NAFLD mice enhanced the expression of HNF4α, thus suggesting the anti-NAFLD role of TFCP via modulating the expression of HNF4α and cytokines. Similarly, a fisetin (a plant flavonoid) treatment suppresses HFD-induced steatosis by upregulating the HNF4α expression and reducing oxidative stress, which further protects the individual from NAFLD progression [24].

In summary, the current study presented evidence that TFCP may attenuate lipid accumulation, inflammation, and oxidative stress in diet-induced NAFLD mice, which suggests the potential benefits of TFCP for the treatment of NAFLD.

## Data Availability

All data included in this study are available upon request by contact with the corresponding author.

## Supplementary Materials

The following supporting information can be downloaded at: https://www.mdpi.com/article/10.3390/cimb44030081/s1. Figure S1: TFCP suppressed hepatic inflammation and oxidative stress in a different group of mice. Mice were fed with MCD or control diet for six weeks, while intragastric administration of TFCP to MCD-fed mice was performed daily. Means ± SEM of results in the graph was obtained using NCD (*n* = 5), MCD diet-fed group (*n* = 5) and MCD+TFCP (*n* = 5).
