# Supplementary material for: Tremella fuciformis Crude Polysaccharides Attenuates Steatosis and Suppresses Inflammation in Diet-Induced NAFLD Mice
Source: Curr Issues Mol Biol. 2022 Mar 3;44(3):1224–34. doi: 10.3390/cimb44030081 (PMC8947202; doi:10.3390/cimb44030081)
Supplement: Supplementary file 1 [file cimb-44-00081-s001.zip › cimb-1475349-supplementary.pdf]

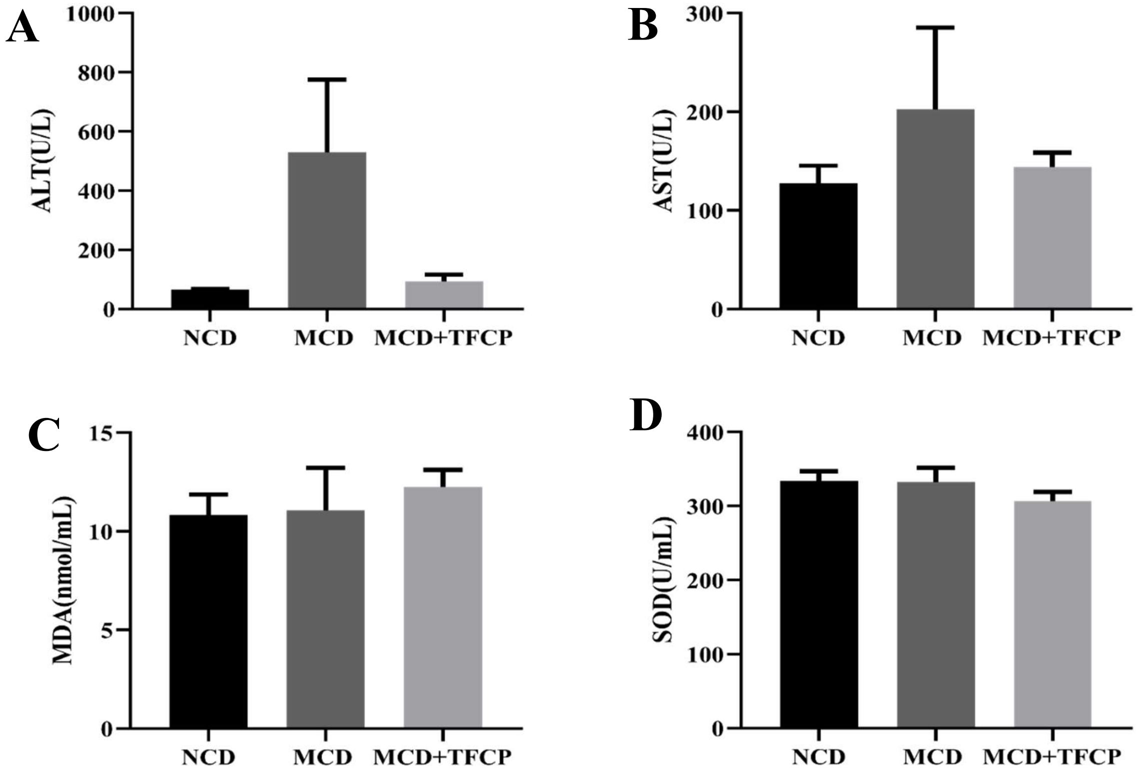


**Figure S1.** TFCP suppressed hepatic inflammation and oxidative stress in a different group of mice. Mice were fed with MCD or control diet for six weeks, while intragastric administration of TFCP to MCD-fed mice was performed daily. Means ± SEM of results in the graph was obtained using NCD ($n$ = 5), MCD diet-fed group ($n$ = 5) and MCD+TFCP ($n$ = 5).
